# A Rare Case of Bicuspid Aortic Valve With Recurrent Endocarditis Complicated by an Aortic Root Pseudoaneurysm

**DOI:** 10.7759/cureus.22161

**Published:** 2022-02-13

**Authors:** Meetham Al Lawati, Mustafa Al-Attraqchi, Srinivasa Sirasanagandla, Salma Khriji, Wijdan Al-Hadhrami, Ahmed Aboul-Azm, Amir Abdelsayed, Rashid Saif AL Umairi

**Affiliations:** 1 Medicine, College of Medicine and Health Sciences, Sultan Qaboos University, Muscat, OMN; 2 Department of Human and Clinical Anatomy, College of Medicine and Health Sciences, Sultan Qaboos University, Muscat, OMN; 3 Department of Cardiothoracic Surgery, National Heart Center, The Royal Hospital, Muscat, OMN; 4 Department of Radiology, The Royal Hospital, Muscat, OMN

**Keywords:** case report, management, infective endocarditis, bicuspid aortic valve, aneurysm

## Abstract

This is a case report of a 35-year-old man who was diagnosed with a bicuspid aortic valve associated with recurrent endocarditis complicated by an aortic root pseudoaneurysm. The pseudoaneurysm is a rare complication. In patients with infective endocarditis (IE), aortic root repair by bovine pericardial patch and subsequent graft infections are considered to be one of the most common risk factors linked to postoperative aortic root pseudoaneurysms. Aneurysms can appear as saccular bulges and are often misdiagnosed as prolapse. The presentation and complicated management are discussed in this case report.

## Introduction

Pseudoaneurysm is a known complication of cardiac surgical procedures. Despite its rare occurrence, it is associated with high morbidity and almost always necessitates a surgical intervention [1]. Bicuspid aortic valve is a common congenital heart lesion seen in 1%-2% of the population. Aortic root pseudoaneurysm following bicuspid aortic valve replacement (AVR) for infective endocarditis (IE) is rare in clinical practice and may cause serious hemodynamic consequences which require immediate reoperation. Graft infections are one of the most common risk factors linked to the postoperative formation of aortic root pseudoaneurysms [2,3]. We present a rare case of recurrent IE episodes in a patient who underwent four operations, including aortic root repair by bovine pericardial patch and AVR, mitral vegitectomy, replacement of infected bovine patch with aortic prosthesis and finally, the removal and replacement of all foreign materials with new aortic homograft.

## Case presentation

A 35-year-old male with no relevant past medical history presented with persistent fever, dyspnea and unintentional weight loss of 15 kilograms over 2-3 months. An echocardiogram was performed, which showed small vegetation attached to the left coronary aortic cusps measuring 12x6mm and aortic root abscess. The whole root abscess was excised from the aortic cusp (non-coronary cusp/right coronary cusp) down to 3mm from the anterior mitral leaflet base with a 2cm width. The defect was repaired by a bovine pericardial patch. Then the AVR was done by mechanical St. Jude valve size 21 HP. Blood culture was negative. He was put on gentamicin, metronidazole, vancomycin, and warfarin until he developed a vancomycin allergy, so he was switched to teicoplanin (1000mg, OD, 28 days). On discharge, he was given both warfarin (5mg, OD) and bisoprolol (2.5mg, OD). On follow-up visits, seven months later, he was doing well, afebrile, and on warfarin. The ejection fraction was 60%, and the peak gradient of AVR was 29/12, while the aortic valve area was 1.8cm^2^.

After about twenty-two months, he presented with an intermittent high-grade fever, chills, rigours, sweating, dry cough, mild upper respiratory tract infection, and right-sided hemiparesis. Two weeks before the emergency presentation, the patient developed an infection of the right molar tooth, underwent treatment, and was discharged on antibiotics. An echocardiogram showed two vegetations in the left atrium; the larger one measured 32x6 mm and it was adherent to the left atrial wall near the aortic root, and the smaller mass measured 9x4 mm attached to the mitral valve. He was diagnosed with recurrent infected vegetation on the mitral valve with minor stroke along with blood culture positive for coagulase-negative staphylococcus. Then, the patient underwent mitral vegetectomy and received gentamicin (1mg/kg TID, two weeks), teicoplanin (1g BID, two days - 1g OD, six weeks), and rifampicin (600mg OD, six weeks). The patient was discharged on warfarin (7mg OD, changed to 5.5mg OD - INR after 5.5mg stabilization ranged between 2.0 - 3.0) and Carvedilol (3.125mg, BID). Four months later, a 2D echocardiography showed mild mitral regurgitation.

After about a year, the patient presented with an infected bovine patch with ventriculo-aortic fistula acting as severe aortic regurgitation although the aortic prosthesis was functioning well and no paravalvular leak, he underwent a second redo-sternotomy with the removal of the aortic prosthesis, and both the destroyed bovine patch and aortic prosthesis were replaced with new ones.

**Figure 1:**
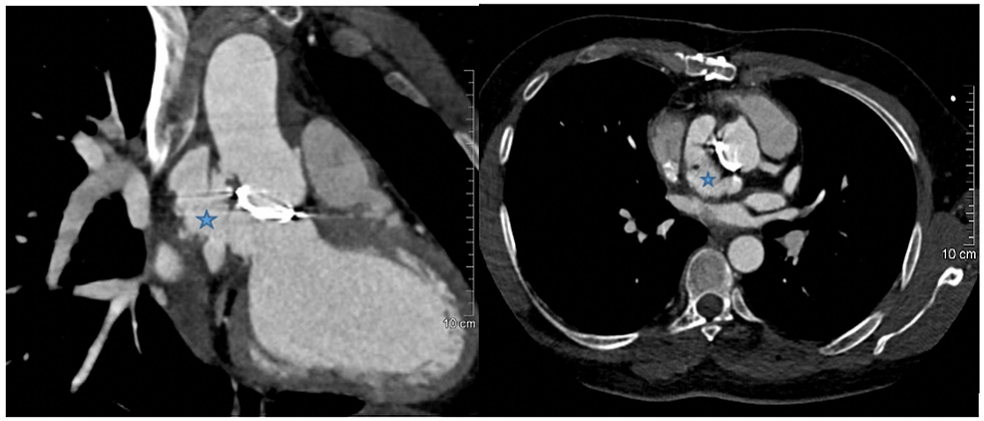
Cardiac computed tomography angiography (CTA) showing a lobulated pseudoaneurysm

After eight months of treatment, the patient presented with persistent low-grade fever for two weeks with watery diarrhea during the first five days of fever, along with intermittent rash in the abdomen and both arms without itching. He continued the oral anticoagulant. Aortic computed tomography angiography (CTA) showed a large aortic root aneurysm located near the left coronary cusps (Figure 1). The aneurysm was originating from the lateral aspect of the left ventricular outflow tract (LVOT) and communicated freely with LVOT through a neck measuring 1.2x1.8 cm in size. As a whole, the aneurysm was measured with dimensions of 5x2.8 cm in size, with no evidence of rupture or collections. His blood culture was positive for Staphylococcus capitis with two other colonies and was tested negative with reverse transcription-polymerase chain reaction (RT-PCR) for COVID-19. Due to the redevelopment of aortic pseudoaneurysm, a new aortic homograft was performed to remove all foreign materials. Figure 1 shows cardiac CTA revealing a lobulated pseudoaneurysm related to the aortic root and connects the ascending aorta to the left ventricle outflow tract (blue star). 

## Discussion

Bicuspid aortic valve has been described as one of the common cardiac congenital defects. Its prevalence is estimated to be between 0.5% and 2% in the general population [4]. Bicuspid aortic valve is associated with the risk of developing many cardiac complications such as aortic stenosis, aortic dissection, and endocarditis [4]. Endocarditis prevalence in bicuspid aortic valve patients is relatively common, especially in the young population with an incidence rate of 1% to 2% [5]. This could be explained based on the bicuspid aortic valve patient’s peculiar aortic histological features. A study by de Sa et al. has demonstrated histological features such as medial cystic necrosis, elastic fragmentation, and changes in the smooth muscle cell orientation in the bicuspid aortic valve patient’s aortic wall [6]. These changes in the media of the aorta predispose subjects to acquire aortic root dilation complications, including IE. Moreover, aortic regurgitation is also one of the accompanying pathologies that take place in bicuspid aortic valve and are also associated with IE patients [5]. Similarly, all the above-mentioned complications were found in the patient’s first admission before his first AVR surgery was performed.

Delahaye et al. reported that the recurrence of IE was 0.3-2.5 per 100 patients per year [7]. The reported risk factors that are associated with IE recurrence include prosthetic endocarditis, periodontitis, and IV drug abuse [8,9]. Furthermore, staphylococcal infection, as a causative agent, has been implicated in the pathogenesis of IE [9]. Staphylococcus aureus and coagulase-negative staphylococci are frequently found to be associated with recurrent IE. Aortic root pseudoaneurysm is a very rare yet significant complication that occurs after a cardiac surgical procedure. Due to their numerous complications, a pseudoaneurysm is considered a life-threatening condition. Some of the complications include the compression of adjacent structures such as the superior vena cava, fistula formation leading to an abnormal connection between structures like the present case where there is a communication between the right lateral aspect of the aortic root and LVOT, rupture of the pseudoaneurysm into the pericardium resulting in cardiac tamponade, and the possibility of thromboembolic events to happen which can cause occlusion of various vessels systematically [10,11].

Although rarely described, the development of pseudoaneurysms is positively linked with the use of bovine patches [12]. Following associations have been laid down to how bovine pericardial patches could lead to the development of pseudoaneurysms. Failure of ingrowth of the tissue surrounding the patch could compromise its integrity leading it to weaken [13]. Morisaki et al.have also reported in an aortic root patch rupture that they have noticed the thinning of the bovine patch adjacent to the vascular prosthesis, which may have been due to frictional forces applied against the prosthetic valvular replacement and the line of suture [12].

## Conclusions

Aortic pseudoaneurysms and aortic-ventricular fistulas are uncommon complications after prosthetic aortic valve endocarditis. The recurrence of prosthetic valve endocarditis itself is a serious condition, and the morbidity rate increases with each recurrence. Echocardiograhy and Cardiac CT are suitable for both the diagnosis and description of pseudoaneurysms and aortic-ventricular fistulas. Although there is a debate regarding the usefulness of the aortic homograft over prosthetic valves in infective endocarditis, the former was a suitable option for our patient since his tissues were expected to be weak due to multiple operations. Further follow-up is required to assess the longevity of the homograft and resolution of IE. Our patient was treated successfully with an elective aortic fistula repair in addition to reoperation of the AVR. At the last available follow-up, the patient was doing well. Due to paucity of data, such anecdotal cases should be reported as it becomes relevant for clinical implication and further research for managing bicuspid aortic valve lesion.
